# “Losing the tombola”: a case study describing the use of community consultation in designing the study protocol for a randomised controlled trial of a mental health intervention in two conflict-affected regions

**DOI:** 10.1186/s12910-015-0032-x

**Published:** 2015-06-02

**Authors:** Leslie Shanks, Claudio Moroni, Isabel Cristina Rivera, Debbie Price, Sifa Banzira Clementine, Giovanni Pintaldi

**Affiliations:** Médecins Sans Frontières-Operational Centre Amsterdam, Amsterdam, Netherlands

**Keywords:** Community consultation, Randomisation, Mental health, Democratic Republic of Congo (DRC), Chechen Republic, Médecins Sans Frontières (MSF), Conflict

## Abstract

**Background:**

Community consultation is increasingly recommended, and in some cases, required by ethical review boards for research that involves higher levels of ethical risk such as international research and research with vulnerable populations. In designing a randomised control trial of a mental health intervention using a wait list control, we consulted the community where the research would be undertaken prior to finalising the study protocol. The study sites were two conflict-affected locations: Grozny in the Chechen Republic and Kitchanga in eastern Democratic Republic of Congo.

**Methods:**

Group discussions with a range of community members were held in both study sites. Facilitators used a prepared set of questions to guide the discussions and to solicit feedback on the value of the research as well as on the study design. Specific questions were asked about enablers and barriers to participation in the research.

**Results:**

Six groups were held in Grozny and thirteen in Kitchanga. The majority of individuals and groups consulted supported the research, and understood the purpose. In Grozny, the main concern raised was the length of the waiting period. Barriers to both waiting and returning for follow up were identified. In Kitchanga, there was a strong reaction against the wait list control and against randomisation. The consultations provided information on unanticipated harms to the community, allowing changes to the study design to mitigate these harms and increase acceptability of the study. It also served to inform the community of the study, and through engaging with them early, helped promote legitimacy and joint responsibility.

**Conclusion:**

Community consultation prior to finalising the study design for a mental health intervention trial in two humanitarian settings proved feasible. Our experience reinforces the importance of community consultation before the study design is finalised and the importance of broad consultation that includes both community leaders and the potential study participants.

**Electronic supplementary material:**

The online version of this article (doi:10.1186/s12910-015-0032-x) contains supplementary material, which is available to authorized users.

## Background

Community engagement is an increasingly common requirement for research initiatives and community consultation is a key component of an overall strategy for community engagement. Community consultation is often used in the scenario of emergency research, where it is a regulatory requirement when individual consent is waived [1]. This has lead to a number of publications on community consultation on the specific issue of waived consent. However community consultation is valuable at all stages of research and is particularly relevant in international research and where research participants are recruited from vulnerable groups. Médecins Sans Frontières’ independent ethics review board stresses the importance of considering community values and culture when doing research, and consulting the community on the research design and implementation [2,3]. The newly proposed Research for Health in Humanitarian Crises (R2HC) framework includes the quality of community engagement as a parameter for the funding committee to consider in reviewing research protocols [4]. Further, it is increasingly recognised that community engagement is not just an ethical requirement, but is critical to the success of the research implementation and of adoption of the findings once completed [5].

Community consultation may take many forms. Examples are surveys including random digit dialling and web-based surveys, focus groups, individual interviews, community information meetings, discussion groups, and community advisory boards [6,7]. A systematic review of results of community consultations done in the setting of research with waived consent, suggests that interactive methodologies (e.g. focus groups, community meetings) are likely to result in higher acceptance rates of the research than less interactive methodologies such as surveys [7].

A recognised challenge in all activities aimed at engaging community is in defining what and who the community is and who has the legitimacy to speak for that community [5,6]. The lack of clear guidance on both methodology for community consultation and issues of community representation highlight the need to document case studies of community consultation in order to demonstrate feasibility, evaluate their impact and develop best practice guidance [5].

Médecins Sans Frontières (MSF) provides medical humanitarian assistance to people in crisis. Psychosocial and mental health interventions are commonly implemented in response to populations affected by conflict and violence. Individual counselling using lay or academically trained counsellors aims to improve functioning and alleviate psychological distress. The approach has been described elsewhere [8] and follows international guidance for intervening in humanitarian settings [9]. In view of the size and importance of our mental health interventions, we planned an effectiveness study with the primary objective of determining if our intervention improves functioning and reduces distressing symptoms. This evaluation followed a review of routinely collected data for the MSF Operational Centre Amsterdam mental health programs, which suggested that the intervention approach required adaption to the context and population served [8]. We were also well aware that, like most psychosocial and mental health interventions used in humanitarian settings, our intervention model had not been formally evaluated for effectiveness.

Since symptoms of mental distress as a consequence of acute exposure to violence often improve with time, use of a control group avoids inflating the impact of the intervention. The challenge comes in designing a suitable control group. Often a wait list control is used in a stepped wedge design, in order to ensure that both groups receive the intervention under study. However in our case, clients are not routinely subjected to wait lists. Nevertheless given that the intervention itself has not yet been shown to be effective, we considered that imposing a wait list could be a reasonable choice. We decided on a design whereby new clients would be randomised to either receive immediate counselling or be on a wait list for three months. In order to ensure those on the wait list would not be denied evidence-based treatment, all those consenting to be in the study would be screened for major psychiatric disorders and suicidal ideation. Either of these conditions would result in immediate medical referral and treatment. In addition, all participants regardless of randomisation would be offered psychological first aid, an intervention that is widely recommended [10–12]. We also planned monthly contact with the wait list controls to ensure there was no new development of high-risk signs and symptoms. There were two sites for the study: an urban setting in Grozny, Chechnya and a rural setting in Kitchanga, North Kivu, Democratic Republic of Congo (DRC).

Given the potential risks with the study design and the additional ethical risks of conducting mental health research in vulnerable populations [13,14] we decided to consult the community prior to finalising the study design and submitting the protocol to ethical review. We defined community consultation as a two-way communication between the researchers and the community. Community discussion groups were chosen as the methodology on the basis of their potential to facilitate exchange of information needed to build comprehension, the ability to reach a broad representation of the diverse community where the studies were to be held and feasibility. The main purpose of the consultation was to receive feedback on the acceptability and feasibility of the study design. In addition, we aimed to inform the community of the study in order to promote participation and ensure understanding of the study in the community.

We describe the methodology we undertook for this community consultation, and report on the results and how they influenced the final study design. Finally we attempt to evaluate the results achieved based on an ethical framework.

## Methods

### Setting

Kitchanga is located in eastern Democratic Republic of Congo in the province of North Kivu. The region has suffered from more than 20 years of violence and conflict along with frequent displacements of the population. The violence is often inter-ethnic, with frequent instances of civilians being targeted and high rates of sexual violence. The town of Kitchanga (population 97,000) is at a crossroads in the frontlines amongst the various armed groups. It hosts two camps for internally displaced persons (IDPs) that are separated on the basis of ethnicity. Literacy rates in DRC are 61.2 % [15] but expected to be much lower in Kitchanga due to the years of conflict. At the time of the consultation, mental health services were delivered in the MSF supported health centres in the IDP camps, as well as at a centre for sexual violence survivors in the town of Kitchanga. Referrals came from medical services and community outreach. MSF has been working in North Kivu to provide free primary and secondary health care since 1991 and has strong networks and acceptance in the community.

Grozny, the capital of the Chechen Republic, has undergone two wars from 1994 to 1996 and 1999 to 2000. The second war was followed by a period of insurgency, which officially ended in 2009. Since then Grozny has been largely re-built and the standard of living has improved for many. However the on-going low-level insurgency means that violence or the threat of violence remains a reality for the population. Literacy rates are not reported for Grozny separately, but the Russian Federation reports a rate of 99.7 % [15]. MSF has been working in Grozny (population 600,000) since 1992, and currently focuses on mental health interventions and a Tuberculosis programme. The outpatient mental health services are based in three hospitals in the Grozny area, and receive referrals from the emergency department as well as self-referrals from the community. Security conditions have only allowed regular presence of international staff in Grozny since early 2012.

### Selection of groups

The community was defined broadly to include medical professionals, key community members (religious and civil leaders, men/women’s groups) as well as the target patient population. In both sites, groups were purposely identified based on this broad definition of community. As MSF keeps a low profile in Chechyna due to security concerns, all groups in Grozny were natural groups drawn from staff and patients attending the three hospitals where MSF works and where the study will take place. In Kitchanga, efforts were made to ensure that key leaders were included as well as members of specific minority groups. Here the composition of groups was also natural, as in the case of the group of religious leaders. Where a natural group did not exist, community health workers identified and asked individuals to be included. Separate groups for men and women were held in Grozny, whereas both gender-specific and mixed groups were formed in Kitchanga. The size of the groups was planned to be 6-8 persons.

### Discussion group methodology

Sessions started with a short introduction to MSF, the mental health programme and the background for the study. Opening questions asked about previous knowledge of MSF, its programs and specifically about the mental health activities. Community informants were then asked for their opinion about the value of the research, and more specifically about the study design and the wait list control. Follow up questions explored ideas about how to motivate individuals on the wait list to remain involved and solicited feedback on specific challenges or barriers seen with executing the research in their community. Probing questions were asked when new themes or ideas arose. Efforts were made to engage all the group members in the discussion. In the one large group in Kitchanga, a vocal minority expressed their opinions and the facilitator attempted to elicit any dissenting views. The topic guide is available as *Additional file 1*. Information and education about the study was shared with participants throughout the discussion to facilitate a two-way exchange of information. The duration for each group discussion was two hours. Resources such as the MSF qualitative methods toolkit were available to the teams to guide methodology [16].

In Kitchanga, three facilitators were present for each group: one leader, one secretary and an observer. The secretary recorded the main comments and opinions of the participants translating from the local language to French. After each group, there was a debriefing with the facilitators and modifications were made to the transcript. At the end of all the sessions, the mental health officer (MHO) along with two counsellors reviewed and categorized the opinions found in the transcription papers. In Grozny, the MHO led the groups while one translator took notes and a second provided simultaneous translation from Russian to English. We did not record the discussions, as there was a risk that using a tape recorder might cause the participants to be uncomfortable given the insecure context of both settings. The analysis was done as a group activity from the written notes using a descriptive, thematic approach derived from the issues raised in the data.

### Ethics statement

The purpose of the consultation was explained to participants, and all participants gave verbal consent to participate. The community consultation took place as part of the ethical requirements for obtaining ethical approval for the trial. As such, specific ethics review board approval was not sought for the work described in this paper.

## Results

In Kitchanga, consultations took place over two weeks in March 2012. There were 13 consultations held with community members in the camps, religious leaders, teachers, the camp committee, local leaders, and local MSF staff with a median of six participants per group (range 5–40). One group of 40 was included in Kit-changa. This was the “kumbanyani’ or neighbourhood chiefs. To split the group would have created distrust therefore all were included. In Grozny, consultations took place over two weeks in February 2013. Six groups were consulted from nurses, social workers, pharmacy staff and medical patients in the hospital. The median number of participants per group was five (range 4–10). The total staff time required for the consultation in Kitchanga was 195 person-hours. In Grozny, it was 36 person-hours.

### On doing the mental health research

#### Kitchanga

There was support expressed for the research and the research question in all groups: ‘*There is a need for an independent evaluation of outcomes of all projects, especially medical ones’*. The minority who did not support the research expressed a number of different concerns. Some felt that the effectiveness of the counselling was already established and saw no need for an evaluation.

*Do your superiors believe that your work does not give fruit that is good? Because I know people who are doing well now after having received your support.*

*It seems you are not sure of what you are doing. We are very happy with what you do and by making the waiting list you will create conflicts in the community.*

Others suggested that the question could be answered without a study by using programmatic data or interviewing old clients. Another objection was linked to distrust of non-governmental organisations (NGOs) and/or MSF. There was a suggestion that the research had a hidden purpose: ‘ …*maybe someone needs a sample for his PhD thesis*’ or ‘*Is there something wrong with the current intervention that they are not telling us?*’

A final concern expressed was the risk of research causing mistrust in the community.

*The questions asked by the researchers must be connected with the illness, if not, it will suggest spying by MSF.*

*MSF must pay attention to the questionnaires. The authorities must also be informed in order that the people did not misinterpret the questions as spying.*

### Grozny

There was general agreement on the value of the research across all groups:

*It is good to do this study and to know about the quality of the work, it can show you what you need to improve or change, what you are doing good or bad.*

*It will be useful to do it. This way you can improve your work if necessary.*

There were no individuals who openly opposed doing the research, however at least one participant did allude to negative reasons for the research when hearing about the control group: ‘*This person thinks only about his own profit, just to say that he/she did this study and he does not care about people’.*

### Control group

#### Kitchanga

The control group proposal provoked the strongest reaction. The need for a control group was not well understood, particularly in the community groups. After explanation, approximately two thirds did agree with the control group, with one third remaining strongly opposed. Community leaders and medical staff were more likely to be supportive than the groups composed of community members. A good explanation was seen as critical: *‘If you give me explanations, I can wait*’. Others stated: ‘*there should be explanations to show that you will not reject those on the waiting list and that they will have follow up’*. It was acknowledged that in any case, each individual would make their own choice to participate or not. The community leader group felt the wait list control was acceptable as long as the exclusion criteria was clear and respected, information shared fully and in an understandable fashion, and the research conducted in a professional manner.

Behind the strong opinions opposing the control group, there were two main themes: the hardship of waiting and the division into groups by random allocation. The three-month time period was felt to be too long but for some, it was deemed impossible to wait even a short period. Parallels were drawn to being ill, and needing medical attention right away: *‘ If I am sick and I want to be treated, I could not go in the tombola*^1^*’*. Many felt that by waiting to start counselling, it would cause their symptoms to become worse.

*Do you think that I could wait the three months without support in the case of your research? I could kill myself or even die in my house.*

Some individuals were sceptical of those agreeing to be in the wait group, expressing that anyone agreeing must be ‘ *faking their symptoms*’ or not really in need.

The randomisation theme centred on the fact that creating two groups would be perceived as a kind of discrimination, even if unintentional. Discrimination or differentiation was seen as a dangerous source of conflicts between neighbours and within families and something that should be avoided in the study. One participant stated simply: ‘*the tombola is going to be complicated*’. Links were made with the current atmosphere of tension and insecurity due to the conflict and the need to avoid even small tensions that could tip the fragile balance.

*Does MSF want to create conflicts in the community?*

*The waiting list will increase the difficulties in the community and a lot of people are going to abandon the programme.*

*The two groups are going to be compared, and those who are on the waiting list will be jealous of those who are getting care.*

In follow up discussions with the counsellors to explore this finding, it was explained that random allocation is linked to a specific perception of random events, namely that chance does not really exist but rather “Fortune” favours its preferred ones. When people are faced with an equal chance of either a good or bad outcome, they tend to accept the challenge, with a secret hope of getting the desired result, without considering the risk of the worst possibility. In the case of a bad result, it confirms the belief that they have been rejected by “Fortune” and are put in disgrace. The mere fact of being put in the control group can therefore psychologically affect the person and affect external perceptions of them.

*If I end up on the waiting list, I may think there is something wrong with me and I could say that you are being biased.*

### Grozny

The initial response of all groups to the control group proposal was that it was too long to ask someone to wait. Some suggested that clients would agree to wait, but then would go see someone else. Others stated that if they are coming for help, they would expect help the same day just as when they come to the hospital and get to see a doctor on the same day. Several people reacted strongly to the idea of a control group, implying that the researchers wanted to humiliate those on the wait list.

*People can think that this way you want to humiliate them. It is showing disrespect for them – if they ask for help and you say to wait for three months, it is like they are mice with which you are doing experiments.*

### How to minimize risk of harm for those in the control group

#### Kitchanga

Client groups emphasised the need for on-going contact for those waiting for counselling. This went further than simple support, and included a strong recommendation to ensure that people in the control group must not feel abandoned or forgotten. This appeared to be linked with the negative connotation of ‘*losing the tombola*’. Means to support those waiting were suggested such as social activities, regular contact or visits at home to ensure the individual on the wait list had some support. Several individuals offered to share what they had learned in counselling with their neighbours as a means of helping them get through the wait.

There was agreement with the exclusion criteria designed to ensure that those requiring urgent treatment were not randomised. It was emphasised that these criteria must be explicit and closely adhered to, in order not to show bias.

### Grozny

Suggestions to limit the risk of waiting and to encourage people to stay in the control group focused on good explanations, though it was felt that this would be more likely to work with more educated individuals. The proposal to give psychological first aid at the first consultation was endorsed: ‘*Probably, it is possible to give one first consultation and ask to wait after that.’*

### Factors influencing participation and retention

#### Kitchanga

The most frequently mentioned point was the importance of giving good and culturally adapted information about the study in order to facilitate collaboration. In nine out of the 11 non-MSF groups, individuals spontaneously offered help in sensitizing and informing others community members about the study.

*We will also help you to explain it to people because we know that the program is important.*

One female participant suggested there was a moral obligation to participate in the study.

*Often we come to you asking for support, you give it to us, and after we feel better. If you want to do the study, we cannot refuse.*

Incentives or compensation for time were rarely proposed as a means to retain study participants despite the heavy reliance of the population on relief goods. When prompted, it was suggested that food rations could be given to compensate for time lost to the study or for being on the wait list. The groups independently agreed that two food rations per month (approximately $10) was an appropriate amount. The risk of people participating only for the sake of the money was raised.

There were mixed feelings about home visits to follow those on the wait list or to remind participants of follow up. Some felt home visits could be an important means of support, while others felt this could be a risk to loss of confidentiality or cause problems with neighbours, depending on the ethnicity of the visitor.

### Grozny

The main practical barriers the groups saw to waiting for three months were family barriers, work and transport or money for transport. Family barriers referred to the need for women to get permission from the male members of their household to participate and to leave the house. Money was suggested in all groups as the main way to get people to participate.

*If you will pay them they will be involved. The more you will pay them, the more motivated they will be.*

The groups did not identify a specific amount of money to compensate participants.

### Influence of the findings on the research

The strong reaction of community members in Kitchanga against randomisation and use of a control group even if mitigated somewhat after further explanation, caused the study team to re-think the study design. An alternative was proposed, that of a control village. This would be possible in a village currently receiving visits from the mobile primary health care team, but not yet receiving a mental health intervention. After a three-month control period, the MH intervention will be introduced and assuming a positive outcome of the study, continued past the study’s completion. This alternative, while less scientifically robust, would eliminate the issue of individual randomisation and allocation into two groups within the same community. As well it would not mean a decline in current standard of care for the control group, but rather introduce a new service to the village.

In Grozny there was no explicit concern about the randomisation or potential ‘discrimination’ between groups that could fuel already existing tensions in the community. All groups raised the difficulty of asking people to wait for counselling. Having received this feedback, the study team decided to review again the wait time and was able to shorten it to two months. This was felt to be more acceptable to the community. As the groups mentioned practical concerns such as loss of time for work and transport costs, it was decided to provide compensation for the time spent on the questionnaires, and to pay travel costs. However as the groups also raised the possibility of undue inducement ---‘*they will come if you pay them but not follow the counselling*’ as one participant put it—it also highlighted the importance of getting the actual amount at the right level to compensate but not induce participation.

The changes prompted by the community consultation were made to both study protocols, and subsequently approved by the MSF Ethics Review Board (ERB). The ERB commented positively on the community engagement activities in the study, and the fact that the researchers adapted the study in response to the feedback. The study also received approval from the Ethical Committee of the Medical Institute of the Chechen State University. It has not yet been submitted to review in DRC, due to an upsurge of violence in the region that has made deferral of the study necessary.

## Discussion

The consultation proved feasible despite the challenging security contexts and provided valuable information to the study team. It raised concerns about the proposed study design, highlighted the importance of good communication about the study, and validated some of the measures in the study designed to protect participants. In particular, the findings from the Kitchanga group on the perception of discrimination, together with the local beliefs around random allocation were important to understand. Neither of these issues had been identified in earlier discussions with the field staff.

A strong message from both Grozny and Kitchanga was the importance of explaining the rationale behind the study design to ensure the community understood this well. There was acceptance that programs need to be evaluated, however particularly for those who believed strongly in the value of the counselling, it was difficult to understand why a control group was needed. Facilitators found that acceptance improved as more explanation was given and was better understood in the more highly educated groups; nevertheless the concept of equipoise for the counselling intervention was challenging to explain and may not have been fully understood. This may have impacted on the findings. This limitation is not unlike community perceptions in other settings where the exposure to clinical trials is higher [17]. However there are clearly additional challenges to explain the study in a cross-cultural setting with variable literacy levels and in communities where familiarity with trials and study designs is minimal.

The challenge of achieving truly informed consent and protecting the autonomy of research participants in vulnerable populations is well known [18]. We did not find evidence that therapeutic misconception, whereby participants mistakenly believe the purpose of the research is to treat their disease rather than to generate generalizable scientific knowledge, was a significant risk in the two communities we consulted. This may be due to the fact that the study design involves a wait list rather than a placebo or comparison intervention, and also the fact that the study intervention will continue to be freely available regardless of participation in the research. It may also be that the questions were not designed to identify this risk specifically.

We were however, made aware of additional risks to our research as MSF, where the blurring of roles between humanitarian actor and researcher can impact on autonomy and capacity to make an informed decision [2,3]. This was expressed by the woman from Kitchanga who indicated that due to all that MSF had given the community, they could not refuse when MSF asked for participation. The right of refusal to consent appeared to be understood by a number of participants, as evidenced by comments that people would refuse to participate or if allocated to the control arm, drop out. There were no references to the possibility that aid would be withheld if someone did not participate. However this woman highlights a sense of moral obligation that would oblige her and others to participate. This is an important issue for researchers to be aware of in conducting the study.

### Evaluation of the results

The validity of the community consultation relies very much on how the community of interest was defined for the study, and how representative the participants of the discussion group were of this community. This is a major theme and debate in the wider literature of community engagement [5,6]. We defined communities on the basis of both geographic area and the target population for our study. As the intervention was aimed at those who had experienced or were experiencing trauma, our target population was broad given the conflict touched all members of the geographic community. We aimed to get feedback from potential clients of the program, as well as the communities’ leaders, whether defined by religious affiliation, administrative boundaries or special interests such as women’s groups. In Kitchanga people belong to multiple groups, and were potentially represented by more than one leader. We found that the groups of leaders were able to approach the questions from the individual level, ‘what is reasonable to ask a study participant’ and also see the wider risks/benefits of the study to the broader community. We included medical staff as we felt they had unique insights into the health needs of the population.

Dickert and Sugarman propose an ethical framework for evaluating the results of community consultations based on what they describe as four universal goals of the consultation [19]. As our objectives for the consultation were primarily ethical, we chose to evaluate our results in this framework. The results are shown in Table 1 and illustrate that all 4 goals were at least partially met for both sites. Less was achieved in Grozny, which can be attributed in part to the limited representation of the community.

**Table 1:** Analysis of results using an ethical framework as proposed by Dickert and Sugarman

Ethical goals	Grozny	Kitchanga
Protection of the community from unforeseen harm	Achieved. Lack of respect to individuals due to waiting list identified as a potential harm. Solutions suggested to mitigate this: clear explanations of trial design, compensation for time, travel, psychological first aid on presentation, etc.	Achieved. The previously unrecognized risk of randomization leading to harm to individuals or the community was identified. Solutions to mitigate potential harms that might accrue from the original design were identified and adopted.
Enhanced benefits to the study participants, the community the research is meant to serve or the community where it took place	Achieved. Wait list period was shortened and compensation to reduce barriers to follow up added, both of which are likely to increase recruitment and retention. A successful trial will ultimately improve chance of benefits to the community through benefits of the research.	Achieved. Design changed to improve uptake of participants and decrease chance of negative individual and community perceptions.
Ethical or political legitimacy	Partially achieved, as consultation prior to finalization of study design allowed the community to have influence on the design. Limited by lack of access to community leaders.	Achieved. As per Grozny, with the added benefit that broad consultation with religious, administration, and political leaders helped achieve political legitimacy.
Shared responsibility	Partially achieved through active engagement with community members in the design and conduct of the trial.	Achieved. Evidence of this was in the spontaneous offer of assistance with the task of informing community members about the trial in 9 out of 11 non-MSF groups.

It is unlikely that we would have achieved these positive results with a less interactive form of consultation such as a survey or information meeting due to the complexity of the research. Compared to the model of Community Advisory Boards (CABs), we were able to achieve broader representation of the community. Our methodology ensured that minority groups and those who have less hierarchy in a community were heard, as we were able to create specific groups for women or other vulnerable groups [20,21]. It also allowed direct consultation with groups of community leaders [21]. It avoided a known tension in CABs between their role in protecting the community and advancing the research [22], as participants had no obligation toward the research itself. However CABs offer significant advantages such as involvement along the trajectory of the research, and improved shared responsibility for the research through direct CAB member involvement in the research implementation. While we were able to achieve some ethical and political legitimacy, CABs may be better placed for the linked objectives of capacity building and community empowerment due to the longitudinal nature of their engagement and the opportunity for training and education of members.

### Limitations

MSF staff led the consultations, which was a strength as they understood MSF well and could clarify any misperceptions. However, this may have biased some of the respondents towards more positive responses as they wished to please, or at least not offend, either MSF or the counsellors personally. An alternative would have been to hire an outside agency to conduct the consultation, however there were few options for this in either Grozny or Kitchanga. In Kitchanga the frequency of negative comments suggests this was not a major limitation, however in Grozny, it is possible that it was more of a concern where public expression of disagreement is less common. For example, one of the Grozny participants asked a translator not to translate his comment, as it was negative towards the research and researchers. Conversely, in Kitchanga, the role of counsellors as facilitators and/or translators may have influenced the interpretation of the results in the opposite direction, as the local counsellors were uncomfortable with the idea of a control group. We attempted to mitigate this by having a MHO present at all groups. The fact that the groups were not recorded may have resulted in missing some information despite our attempt to compensate for this by having three facilitators in each group. Finally, our methodology was not that of formal focus groups but rather group discussions that promoted interactive communication. This may have affected the validity of some of our conclusions.

## Conclusion

Community consultation using an interactive methodology prior to finalising the study design for a mental health intervention trial in two humanitarian settings provided valuable information on unanticipated harms to the community. The consultation resulted in changes in the study design while endorsing other aspects of the study. It served to inform the community of the study, and through engaging with them early, helped promote legitimacy and joint responsibility. This experience reinforces the importance of community consultation before the study design is finalised, highlights the importance of broad community representation and finally suggests that community consultation can reduce the risks to autonomy posed by researchers from humanitarian agencies working with vulnerable populations.

## Additional file

Additional file 1:
**Topic List for Discussion Groups.**

## Abbreviations

CAB: Community Advisory Board
DRC: Democratic Republic of Congo
ERB: Ethics Review Board
IDP: Internally displaced person
MSF: Médecins Sans Frontières
MHO: Mental Health Officer
NGO: Non-governmental organisation
R2HC: Research for Health in Humanitarian Crises

## Footnotes

1 A tombola refers to a specific type of lottery whereby tickets are put in a rotating drum to be drawn for prizes. Here participants used the term generically to refer to a lottery or draw.

## References

[1] US Department of Health and Human Services Code of Federal Regulation (1996). Protection of Human Subjects: informed consent and waiver of informed consent requirements in certain emergency research: final rules 21 CFR Part 50.24 and 45 CFR Part 46.101. Fed Reg.

[2] Schopper D, Upshur R, Matthys F, Singh JA, Bandewar SS (2009). Research Ethics Review in Humanitarian Contexts: The Experience of the Independent Ethics Review Board of Médecins Sans Frontières. PLoS Med.

[3] Nathan Ford N, Mills EJ, Zachariah R, Upshur REG (2009). Ethics of conducting research in conflict settings. Confl Health.

[4] Curry DR, Waldman RJ, Caplan AC. An Ethical Framework for the development and review of health research proposals involving humanitarian contexts. Project Final Report, January 2014. Enhancing Learning and Research for Humanitarian Assistance. Available at: http://www.elrha.org/wp-content/uploads/2015/01/FINAL-R2HC-Ethical-Framework_Final-Report_24-January-2014_0.pdf. Accessed March 9, 2014

[5] Tindana P, Singh JA, Tracy CS, Upshur REG, Daar AS (2007). Grand Challenges in Global Health: Community Engagement in Research in Developing Countries. PLoS Med.

[6] Participants in the Community Engagement and Consent Workshop (2013). Kilifi, Kenya, March 2011. Consent and community engagement in diverse research contexts: reviewing and developing research and practice. J Empir Res Hum Res Ethics.

[7] Fehr A, Pentz RD, Dickert ND. Learning from experience: A systematic review of community consultation acceptance data. Ann Emerg Med. 2015 Feb;65(2):162-71.e3. doi:10.1016/j.annemergmed.2014.06.023. 10.1016/j.annemergmed.2014.06.023 25085547

[8] Shanks L, Ariti C, Siddiqui R, Pintaldi G, Venis S, de Jong K, Denault M (2013). Counselling in humanitarian settings: a retrospective analysis of 18 individual-focused non-specialised counselling programmes. Confl Health.

[9] IASC (2010). Reference Group for Mental Health and Psychosocial Support in Emergency Settings: Mental health and psychosocial support in humanitarian emergencies: what should humanitarian health actors know?.

[10] Inter-agency standing committee (IASC) (2007). IASC guidelines on mental health and psychosocial input support in emergency situations.

[11] World Health Organisation (2011). War Trauma Foundation and World Vision International. Psychological first aid: Guide for field workers.

[12] Fox JH, Burkle FM, Bass J, Pia FA, Epstein JL, Markenson D (2012). The Effectiveness of Psychological First Aid as a Disaster Intervention Tool: Research Analysis of Peer-Reviewed Literature From 1990-2010. Disaster Med Public Health Prep.

[13] MacKenzie C, MCDowell C, Pittaway E, Beyond Do No H (2007). The Challenge of Constructing Ethical Relationships in Refugee Research. J Refugee Stud.

[14] DuBois J, Bailey-Burch B, Bustillos D, Campbell J, Cottler L (2011). Ethical issues in mental health. Curr Opin Psychiatry.

[15] United Nations Development Programme (2014). Human Development Report.

[16] Bricki N, Green J. A guide to using qualitative methods. 2007. London: Médecins Sans Frontières. Available at: http://fieldresearch.msf.org/msf/handle/10144/84230. Accessed Feb 22, 2014.

[17] Kerr C, Robinson E, Stevens A, Braunholtz D (2004). Edwards Set al., Randomisation in trials: do potential trial participants understand it and find it acceptable?. J Med Ethics.

[18] Siriwardhana C, Adikari A, Jayaweera K, Sumathipala A (2013). Ethical challenges in mental health research among internally displaced people: ethical theory and research implementation. BMC Med Ethics.

[19] Dickert N, Sugarman J. Ethical Goals of Community Consultation in Research. Am J Public Health Jul 2005, 95, 7; ABI/INFORM Global pg. 1123 10.2105/AJPH.2004.058933 PMC1449329 15983268

[20] Maung Lwin K, Cheah PY, Cheah PK, White NJ, Day NPJ (2014). Motivations and perceptions of community advisory boards in the ethics of medical research: the case of the Thai-Myanmar border. BMC Med Ethics.

[21] Kamuya DM, Marsh V, Kombe FK, Geissler PW, Molyneux SC (2013). Engaging communities to strengthen research ethics in low-income settings: selection and perceptions of members of a network of representatives in coastal Kenya. Dev World Bioeth.

[22] Reddy P, Buchanan D, Sifunda S, James S, Naidoo N. The role of community advisory boards in health research: Divergent views in the South African experience. SAHARA J. 2010;7(3):2–8. 10.1080/17290376.2010.9724963 21409299

