# Supplementary material for: “Losing the tombola”: a case study describing the use of community consultation in designing the study protocol for a randomised controlled trial of a mental health intervention in two conflict-affected regions
Source: BMC Med Ethics. 2015 Jun 2;16:38. doi: 10.1186/s12910-015-0032-x (PMC4450849; doi:10.1186/s12910-015-0032-x)
Supplement: Additional file 1: — Topic List for Discussion Groups. [file 12910_2015_32_MOESM1_ESM.docx]

Additional File 1: Topic List for Discussion Groups

- Introduction: Provide information about the study and the benefit for international mental health interventions
- What do they know about MSF in Kitchanga/Grozny and its history
- What do they know about mental health activities provided by MSF
- What is their opinion about collecting information on outcomes of MH activities as part of a scientific study on effectiveness of MSF’s approach
- Do they think it is possible to ask patients to wait 3 months for counselling and if yes how to ask?
- Input on how to keep the control group motivated and continue to be involved
- Input on what is a reasonable incentive for participating in the study
